# Carcinome vesiculaire sur thyroïde linguale: cas rare

**DOI:** 10.11604/pamj.2017.26.29.11482

**Published:** 2017-01-23

**Authors:** Said Anajar, Mohammed Moutaa Tatari, Jawad Hassnaoui, Sami Rouadi, Reda Abada, Mohammed Roubal, Mohammed Mahtar

**Affiliations:** 1 Service ORL et Chirurgie Cervico-faciale, Hôpital 20 Août, Casablanca, Maroc

**Keywords:** Thyroïde linguale, carcinome, thyroïde, ectopique, Lingual thyroid, carcinoma, thyroid, ectopic

## Abstract

La thyroïde linguale est secondaire à l'absence de migration de l'ébauche thyroïdienne. Cette dernière se développe sur place, c'est-à-dire au niveau du foramen caecum. Elle peut représenter le seul tissu thyroïdien retrouvé, ou être en association avec une thyroïde normale. La survenue d'un carcinome sur une thyroïde linguale constitue une situation exceptionnelle avec seulement une trentaine de cas décrits dans la littérature. Nous rapportons a travers notre observation un cas de carcinome vésiculaire survenu sur thyroïdien lingual.

## Abstract (en)

Lingual thyroid is secondary to the absence of migration of the thyroid diverticulum. The latter develops locally, that is, at the level of the foramen caecum. It may be the only thyroid tissue present, or it may be associated with a normal thyroid. The occurrence of a lingual thyroid carcinoma is rare accounting for about thirty cases described in the literature. This study reports a case of vesicular carcinoma in lingual thyroid.

## Introduction

La thyroïde ectopique est une anomalie embryologique rare caractérisée par la présence de tissu thyroïdien en dehors de sa situation normale. Elle résulte d'une anomalie survenant lors de la migration laissant une partie ou la totalité du tissu thyroïdien le long du tractus thyréoglosse. La thyroïde linguale (TL) est la forme la plus fréquemment rencontrée. Elle peut représenter le seul tissu thyroïdien retrouvé chez le patient, ou être en association avec une thyroïde normale. Le tissu ectopique peut présenter toute lésion affectant la glande thyroïde, y compris les carcinomes qui représentent moins de 1% des néoplasies thyroïdiennes. Les carcinomes vésiculaires représentent le tiers des cas rapportés [1]. Le pronostic des carcinomes sur TL (CTL) est encore peu connu, étant donné le faible nombre de cas rapporté et l'hétérogénéité des attitudes thérapeutiques. Le but de ce travail est de décrire les modalités de prise en charge d'un cas de carcinome vésiculaire survenu sur parenchyme thyroïdien lingual.

## Patient et observation

**Figure 1:**
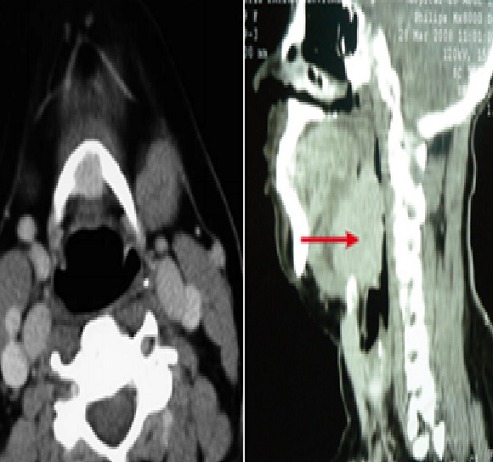
TDM cervicale montrant un processus tissulaire étendu vers la base la langue

**Figure 2:**
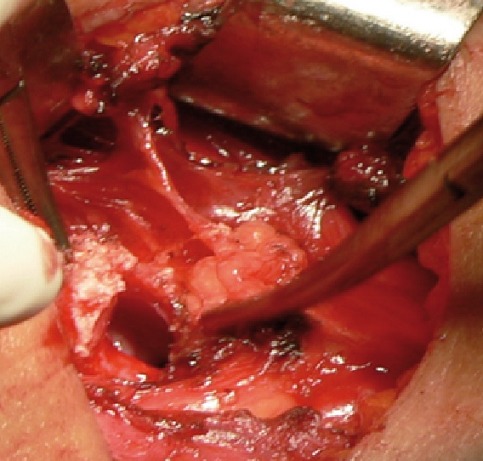
Aspect per opératoire à la dissection

Il s'agit d'une patiente âgée de 47 ans sans antécédents pathologiques particuliers qui présentait une dysphagie évoluant depuis 7 mois. L'examen clinique était sans particularités. L'échographie cervicale a montré une masse tissulaire du plancher buccal. Une nasofibroscopie a mis en évidence un bombement au niveau de la vallecule droite. Le scanner cervico-facial a montré un processus d'allure tumorale centré sur l'épiglotte, s'étendant vers la base de langue et bombant dans la lumière de l'oropharynx (Figure 1). La glande thyroïde n'a pas été individualisée sur les images cervicales. Une scintigraphie thyroïdienne a montré un foyer de fixation haut situé en sus hyoïdien surmonté d'un deuxième foyer. Une biopsie au cours d'une laryngoscopie directe a précisé la nature thyroïdienne du prélèvement. L'indication opératoire étant posée. La dissection cervicale médiane (Figure 2) a découvert une masse d'allure thyroïdienne composée de deux foyers. L'examen histologique a confirmé sa nature thyroïdienne avec a présence en son sein un foyer de carcinome vésiculaire. La patiente a bénéficié d'une irathérapie en post opératoire avec une scintigraphie corps entier montrant une carte isotopique blanche. L'examen cervical de contrôle trouve une loge thyroïdienne libre sans adénopathies.

## Discussion

L'ébauche thyroïdienne apparaît à la fin de la troisième semaine du développement embryonnaire. La position anatomique finale est atteinte autour de la 4ème semaine. Une aberration survenant au cours de la migration peut laisser une partie ou la totalité du tissu thyroïdien à la base de la langue ou à une autre position le long du tractus thyréoglosse [1]. La prévalence de la thyroïde ectopique est de 1100 000 et sa transformation cancéreuse peut se produire dans 1-3% des cas [1]. La pathogénie de l'ectopie thyroïdienne est encore non élucidée [2]. Certains pensent que des immunoglobulines maternelles dirigées contre les antigènes thyroïdiens seraient à l'origine de cet arrêt de migration [3]. La transformation carcinomateuse de la thyroïde linguale est très rare avec seulement une trentaine de cas répertoriés dans la littérature [4, 5]. Le type vésiculaire est le plus fréquent [6]. Cliniquement elle peut se manifester par une dysphonie une dysphagie ou une dyspnée [7]. La survenue d'une douleur cervicale ou d'un saignement doit faire craindre une transformation carcinomateuse, qui survient dans 1 à 3% des cas selon certains auteurs [8]. La cytoponction est utile pour le diagnostic différentiel entre thyroïde linguale bénigne et carcinome [9], Les imageries utilisées pour l'exploration d'une thyroïde linguale sont la tomodensitométrie, l'imagerie par résonance magnétique (IRM) et la scintigraphie utilisant l'iode 123, ou le technétium 99m (Tc99m) [10]. La fixation du radionucléotide (Iode 131 ou Tc99m) au niveau de la base de la langue confirme la nature thyroïdienne du tissu ectopique. Sur le plan thérapeutique, la prise en charge en cas de carcinome différencié consiste en une résection chirurgicale suivie d'un traitement complémentaire par irathérapie, en fonction du résultat du balayage postopératoire et du taux initial de la thyroglobuline [9]. La voie transcervicale transhyoïdienne utilisée pour notre patiente permet un plus large champ opératoire que la voie trans-orale. Le suivi est effectué par l´examen clinique, la mesure de la TSH, la thyroglobuline (Tg) couplée à la détermination d´anticorps anti-Tg, scintigraphie du corps entier et de l'échographie cervicale [9].

## Conclusion

Le diagnostic de thyroïde ectopique est généralement confirmé par l'imagerie qui évalue ses rapports avec les structures environnantes. La survenue d'un carcinome vésiculaire sur une thyroïde linguale est une situation très rare.
